# Liraglutide 3.0 mg for the management of insufficient weight loss or excessive weight regain post‐bariatric surgery

**DOI:** 10.1111/cob.12323

**Published:** 2019-06-10

**Authors:** Sean Wharton, Jennifer L. Kuk, Magdalena Luszczynski, Elham Kamran, Rebecca A. G. Christensen

**Affiliations:** ^1^ The Wharton Medical Clinic Toronto Ontario Canada; ^2^ Department of Kinesiology and Health Science York University Toronto Ontario Canada

**Keywords:** bariatric surgery, liraglutide 3.0 mg, pharmacologic therapy, weight loss

## Abstract

To assess the effectiveness of liraglutide 3.0 mg in post‐bariatric surgery patients, and to determine whether this would differ based on the type of bariatric surgery. One hundred seventeen post‐bariatric surgery patients from the Wharton Medical Clinic were analysed. Changes in weight while taking liraglutide 3.0 mg were examined for all patients, and by three types of bariatric surgery—Roux‐en‐Y gastric bypass, gastric banding and gastric sleeve. Patients primarily underwent Roux‐en‐Y gastric bypass (n = 53, 45.3%) or gastric banding (n = 50, 42.7%). Over 7.6 ± 7.1 months taking liraglutide 3.0 mg, patients lost a statistically significant amount of weight (−6.3 ± 7.7 kg, *P* < .05) regardless of the type of surgery they had (*P* > .05). This decrease in weight remained significant after 1‐year of taking liraglutide 3.0 mg (*P* < .05). Nausea was the most prevalent side effect, reported by 29.1% patients. While options for excess weight management in post‐bariatric surgery patients are limited, results of this study suggest that post‐bariatric surgery patients can lose a significant amount of weight while taking liraglutide 3.0 mg regardless of the type of surgery they had. Further, similar to non‐surgical populations, post‐bariatric surgery patients taking liraglutide 3.0 mg may experience gastrointestinal side effects such as nausea and can continue to lose weight up to 1 year.

## 1 INTRODUCTION

Bariatric surgery is considered the gold standard treatment for weight loss in patients with obesity and complications of obesity, based on superior long‐term weight loss outcomes, and improvements in comorbidities compared to lifestyle and pharmaceutical interventions.^1^ Despite the dramatic weight loss achieved with bariatric surgery (30%‐45% weight loss^2^), 10% to 20% of bariatric surgery patients will regain a significant amount of the weight they had lost,^3^ which can be defined as a patient regaining 10% to 25% of excess weight or total weight lost.^4^ Regardless, as greater number of patients have surgery, the concern of weight regain post‐surgery has become significant.

In 2015, liraglutide 3.0 mg was approved for the treatment of excess weight in Canada.^5^ In non‐surgical populations, liraglutide 3.0 mg has been found to be associated with significant reductions in appetite and weight.^6^ However, as surgical patients were excluded from phase III weight management pharmaceuticals trials, the effectiveness of liraglutide 3.0 mg in this population is unknown. Thus, the objective of the current study was to examine the weight loss associated with the use of liraglutide 3.0 mg in post‐bariatric surgery patients.

## 2 METHODS

The Wharton Medical Clinic (WMC) are referral‐based secondary care clinics for weight and diabetes management located across southern Ontario. WMC provides care in accordance with the policies in the Canadian Clinical Practice Guidelines for the treatment of obesity in adults and children and National Institutes of Health Clinical Guidelines on the identification, evaluation and treatment of overweight and obesity in adults. All services provided to patients by the WMC are covered by the Ontario Health Insurance Program. Patients were informed that their participation or lack thereof would not hinder their medical treatment and provided written consent for access to their medical records to conduct research. All methods were approved by the York University Institution Review Board in accordance with the Tri‐Council Ethical Conduct for Research Involving Humans, version 2.

There was a total of 649 post‐bariatric surgery patients referred to WMC for weight management and who consented for their data to be used for research. Of these, 174 patients were prescribed liraglutide 3.0 mg for weight management. Patients were excluded if they did not initiate liraglutide 3.0 mg, resulting in 117 patients for analysis. Of the 57 patients who did not initiate liraglutide 3.0 mg, 10 were lost to follow‐up, and three initiated Contrave (an oral weight management medication). The remaining 44 patients participated in the lifestyle intervention at the clinic without adjunctive therapies. Over the 14.7 ± 24.0 months these patients attended the clinic, they lost 2.1 ± 9.0 kg (*P* > .05). The reason for not initiating liraglutide 3.0 mg was recorded for only six patients, and all stated it was due to cost.

### 2.1 WMC protocol

WMC procedures have been previously discussed in greater detail elsewhere.^7^ During the initial visit to the clinic, patients complete a comprehensive intake questionnaire on demographics, and medical and weight management history. Anthropometric measurements are done on all patients by trained technicians. Height is measured by a wall‐mounted tape to the nearest 0.1 cm (McArthur Medical Sales, Inc., Ontario), and weight which is measured using a digital scale to the nearest 0.1 kg (Itin Scale Co, Inc., New York). Subsequently, patients attend an introductory educational session about WMC's policies and procedures, followed by an appointment with a bariatric educator and medical doctor. The bariatric educator provides weight management support by establishing dietary changes and exercise regimes. The medical doctor examines and reviews the patient's medical history, discusses pharmaceutical management or surgical intervention where clinically indicated. Patients who choose to initiate liraglutide 3.0 mg are recommended to follow a dosing schedule starting at 0.6 mg that is titrated 0.6 mg/mL until reaching the maximum clinical dosage of 3.0 mg, but this may vary in clinical practice.^8^ The physician orders blood work, resting metabolic rate test and other relevant tests for each patient.

Patients are given a meal plan at their third visit which includes a caloric deficit of approximately 500 kcal/day based on their daily energy expenditure. The bariatric educator reviews the meal plan with the patient and discusses food alternatives and snacks. The patient also meets with the physician who examines the patient, reviews the updated anthropometric measurements, and monitors comorbidities and pharmaceuticals.

Following the initial three visits, patients are encouraged to attend monthly appointments at the clinic; however, patients may attend more often if they desire. At all subsequent appointments patients meet with a physician and/or a bariatric educator for continued weight management support.

Weight (kg), height (cm), sex (female/male), age (years), treatment time (months) and type of bariatric surgery (ie, adjustable gastric banding (AGB), Roux‐en‐Y gastric bypass (RNYGB) and gastric sleeve), maximum dose achieved on liraglutide 3.0 mg, side effects, and reason for discontinuation were extracted from electronic medical records. Clinically significant weight loss was defined as >5% decrease in body weight as per clinical guidelines.^9^, ^10^ Percent change in weight from lowest weight post‐bariatric surgery to initiating liraglutide 3.0 mg was calculated as ([(maximum weight change post‐bariatric surgery)/(weight change from lowest post‐bariatric surgery weight to initiation of liraglutide 3.0 mg)] × 100). Patients reported more than 1 side effect, thus the percent of adverse events were calculated based on the total number of patients included in the study (ie, [(number of patients reporting the side effect)/117] × 100). Maximum clinical dose was dichotomized as achieving the recommended clinical dose of 3.0 mg (yes/no). All weight variables were measured by trained technicians with the exception of weight prior to bariatric surgery and the maximal weight lost with bariatric surgery which was self‐reported by patients.

### 2.2 Statistical analysis

Continuous variables are reported as mean ± SD and categorical variables as frequency (prevalence). Independent *t* test was undertaken to compare weight change between patients who did and did not achieve the recommended clinical dose of liraglutide 3.0 mg. One‐way analysis of variance (ANOVA) was used to examine differences in age, body mass index (BMI), treatment time, percent changes in weight, and the side effect nausea and repeated measures ANOVA was used to examine absolute changes in weight based on the type of bariatric surgery patients had underwent. Least‐square difference post‐hoc was used where indicated. Differences in sex and the proportion of individuals achieving ≥5% and ≥10% weight loss based on the type of bariatric surgery were assessed using chi‐square analysis. Changes in weight were compared in 2‐month increments from 1‐month post‐initiation up to 1 year based on the type of bariatric surgery using proc mixed. Changes in weight loss were also compared using proc mixed for patients who were persistent on liraglutide 3.0 mg for ≤2 months, >2 to ≤4 months, and >4 months using proc mixed. All analyses were conducted using SAS version 9.4 (SAS Institute). A *P*‐value ≤.05 was considered statistically significant.

## 3 RESULTS

One hundred seventeen patients were included in the analysis. Patients were primarily female (87.2%) and were 51.2 ± 9.4 years of age. The majority of patients underwent RNYGB (45.3%) or AGB (42.7%). Prior to initiating bariatric surgery, patients had a BMI of 49.7 ± 12.1 kg/m^2^ and lost 40.7 ± 25.0 kg (28.8%) with surgery. Prior to initiating liraglutide 3.0 mg, patients had regained weight 21.2 ± 16.9 kg (58.6%) of their weight loss with an average BMI of 42.5 ± 9.6 kg/m^2^. Patients initiated liraglutide 3.0 mg approximately 8 years (7.8 ± 5.7 years, interquartile range: 4‐10 years) post‐bariatric surgery. Over 7.6 ± 7.1 months taking liraglutide 3.0 mg, patients lost 5.5 ± 6.2% (6.3 ± 7.7 kg, *P* < .05) of their body weight.

**Figure 1:**
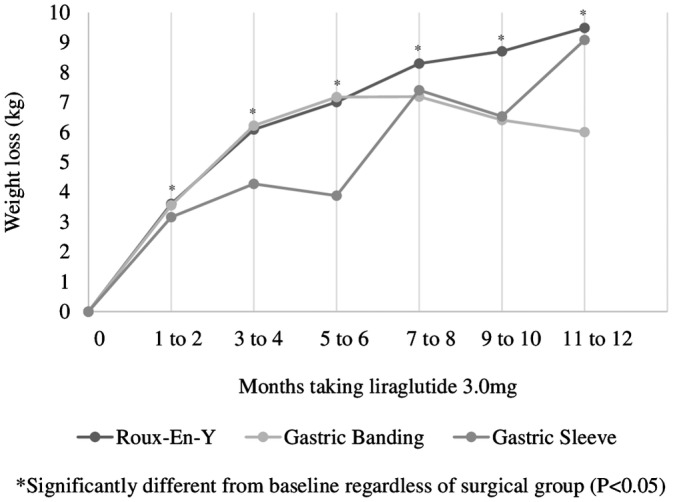
Weight loss while taking liraglutide 3.0 mg over time by type of bariatric surgery

Patients who had undergone RNYGB had a lower BMI prior to initiating liraglutide 3.0 mg than patients who had received a AGB or sleeve gastrectomy (*P* < .05, Table 1). Regardless of the surgery, patients who took liraglutide 3.0 mg lost a similar amount of weight (*P* > .05). Patients experienced a significant weight loss as early as 1 to 2 months post‐initiation of liraglutide 3.0 mg, which remained significant up to 1 year of taking the medication (*P* < .05) regardless of the type of bariatric surgery they had (*P* > .05, Figure 1). Further, on average those persistent on liraglutide 3.0 mg lost significantly more weight by 11 to 12 months post‐initiation than at 5 to 6 months (2.0 ± 7.4 kg, *P* = .004). As expected, patients who discontinued liraglutide 3.0 mg within 4 months lost less weight early in the treatment as compared to patients who remained on the drug for greater than 4 months (data not shown, *P* < .05).

**Table 1:** Patient characteristics by type of bariatric surgery

Variable	Roux‐en‐Y bypass	Gastric band	Gastric sleeve
Sample size (n)	53	50	14
Age (y)	49.9 ± 9.1	52.5 ± 9.5	51.4 ± 10.3
Men (n, %)	3 (5.7)^a^	8 (16.0)	4 (28.6)
Pre‐bariatric surgery BMI (kg/m^2^)	50.8 ± 11.2	47.6 ± 13.1^e^	52.2 ± 11.9
Maximum weight change post‐bariatric surgery (kg)	−51.6 ± 23.5^a^^,^ b	−29.8 ± 23.3^e^	−34.7 ± 19.5
Weight change from lowest post‐bariatric surgery weight to initiation of liraglutide 3.0 mg (kg)	19.0 ± 13.5	25.4 ± 20.4^e^	15.8 ± 14.1
Weight change from lowest post‐bariatric surgery weight to initiation of liraglutide 3.0 mg (%)^c^	44.8 ± 54.9^a^	80.0 ± 79.7^f^	48.4 ± 31.7^g^
Preliraglutide BMI (kg/m^2^)	39.0 ± 7.0^a^^,^ b	45.4 ± 11.0	45.4 ± 9.6
Weight change on liraglutide 3.0 mg (kg)	−7.1 ± 8.7^d^	−6.0 ± 7.2^d^	−4.5 ± 4.5^d^
Weight change on liraglutide 3.0 mg (%)	−6.6 ± 7.1	−4.9 ± 5.6	−3.6 ± 3.0
Attained 5% weight loss (n, %)	25 (47.2)	19 (38.0)	5 (35.7)
Attained 10% weight loss (n, %)	13 (24.5)^a^	6 (12.0)	0 (0.0)
Treatment time (mo)	8.0 ± 7.6	6.8 ± 6.7	8.6 ± 7.3
Reported nausea with liraglutide 3.0 mg (n, %)	15 (28.3)	12 (24.0)	5 (35.7)

a Significantly different from gastric sleeve (*P* < .05).

b Significantly different from gastric band (*P* < .05).

c Calculated as [(maximum weight change post‐bariatric surgery)/(weight change from lowest post‐bariatric surgery weight to initiation of liraglutide 3.0 mg)] × 100.

d Significant change from weight prior to initiating liraglutide 3.0 mg (*P* < .05).

e n = 45.

f n = 40.

g n = 13.

More than half of the participants (n = 73 of 117, 62.4%) achieved the recommended maximum clinical dose of 3.0 mg. Of the participants (n = 94 of 117, 80%) that had taken liraglutide 3.0 mg for long enough (ie, >1 month) to titrate to the recommended maximum clinical dose, 25.5% (n = 24 of 94) did not achieve the recommended maximum clinical dose. The proportion of patients who did not achieve the recommended maximum clinical dose of 3.0 mg was similar regardless of type of bariatric surgery (*P* = .29). Patients who were persistent on liraglutide 3.0 mg for more than 1 month (n = 94 of 117) lost a similar amount of weight whether or not they achieved the recommended maximum clinical dose (7.4 ± 6.9 vs 6.8 ± 11.4, *P* = .81).

A total of 92 side effects were reported among 59 of 117 patients (50.4%, Table 2). Gastrointestinal side effects were the most prevalent, with the top three most frequently reported side effects being nausea (n = 34 of 117, 37.0%), constipation (n = 13 of 177, 14.1%) and diarrhoea (n = 8 of 117, 8.7%). A similar proportion of patients reported nausea as a side effect from taking liraglutide 3.0 mg regardless of the type of surgery they had undergone (*P* = .96). Other side effects were rare and were generally mild with the exception of one patient developing pancreatitis (0.9%).

**Table 2:** Side effects for liraglutide 3.0 mg

Symptom	n (%)^a^
Nausea	34 (29.1%)
Constipation	13 (11.1%)
Diarrhoea	8 (6.8%)
Fatigue	7 (6.0%)
Headache	4 (3.4%)
Rash	4 (3.4%)
Indigestion	3 (2.6%)
Vomiting	3 (2.6%)
Dry mouth	3 (2.6%)
Bloating	2 (1.7%)
Sweating	2 (1.7%)
Other^b^	9 (7.7%)

a Percent calculated as [(number of patients reporting the side effect)/117] × 100.

b Other includes abdominal pain (n = 1), bruising (n = 1), decreased glomerular filtration rate (n = 1), depression (n = 1), flu‐like symptoms (n = 1), heartburn (n = 1), hot flashes (n = 1), gas (n = 1) and pancreatitis (n = 1).

Over the observation period, just over one‐third of patients were persistent on liraglutide 3.0 mg (n = 43, 36.8%). Of the additional 74 patients, 62.2% (n = 46) of patients were lost to follow‐up, and therefore we cannot ascertain whether or not they discontinued taking liraglutide 3.0 mg. Twenty‐eight participants reported discontinuing liraglutide 3.0 mg, and details regarding the reason for discontinuation was captured for 20 participants. The most commonly reported (n = 10 of 20, 50%) reason for discontinuing liraglutide 3.0 mg was lack of effectiveness for weight management. Other reasons for discontinuing liraglutide 3.0 mg include cost (n = 7 of 20, 35%) and having an adverse event (n = 3 of 20, 15%), which include an allergic reaction (n = 2 of 3), and pancreatitis (n = 1 of 3).

## 4 DISCUSSION

Options for the management of excessive weight regain or insufficient weight loss post‐bariatric surgery are limited, and with the increase in the number of patients receiving bariatric surgery, there is a pressing need for additional treatment options. Fortunately, results of this study suggest that post‐bariatric surgery patients taking liraglutide 3.0 mg can lose a significant amount of weight. Indeed, significant reductions in weight were observed shortly after initiating liraglutide 3.0 mg and up to 1‐year post‐initiation of the medication regardless of the type of bariatric surgery patients had. Similar to what is observed in non‐post‐surgical populations, post‐bariatric surgery patients report side effects from the medication that are primarily gastrointestinal in nature and can continue to lose weight up to 1‐year post‐initiation of liraglutide 3.0 mg.

Treatment options for patients with insufficient weight loss or excessive weight regain following bariatric surgery are limited to repair of post‐surgical issues, conversion (typically from a restrictive procedure to one that incorporates a greater degree of malabsorption), endoscopic therapies, and/or reintroduction of lifestyle management techniques. However, revision and conversion surgeries have higher rates of complications than the initial procedure itself.^11^ Thus, pharmacotherapy may provide a safer alternative for weight management in this patient population. Currently, there is no weight management pharmaceutical approved for use in patients who have had bariatric surgery as having had bariatric surgery is often an exclusion for non‐surgical weight loss trials. However, research on the off‐labelled prescription of weight management pharmaceuticals in post‐bariatric surgery patients have reported significant decrease in weight post‐initiation of these agents.^12^, ^13^, ^14^ Consistent with these findings, post‐bariatric surgery patients in the current study taking liraglutide 3.0 mg lost a significant amount of weight. This may suggest that the use of weight management pharmaceuticals may be an effective treatment to address excessive weight regain or insufficient weight loss in post‐bariatric surgery patients.

Surgeries that remove a portion of the stomach (eg, RNYGB and gastric sleeve) are associated with increase in glucagon‐like peptide‐1 (GLP‐1) levels, which have been thought to in part contribute to sustained weight loss post‐surgery.^15^, ^16^ Despite these well‐known differences due to the type of bariatric surgery, few papers have examined the differences in weight loss by surgery type for those taking weight management pharmaceuticals. The studies which have primarily examined the use of phentermine, phentermine/topiramate, and naltrexone/bupropion, and report no differences for patients who had undergone RNYGB and AGB. However, individuals who had undergone gastric sleeve lost less weight than those who had undergone RNYGB and AGB.^13^, ^17^ Consistent with previous literature, we did observe similar reductions in weight while taking liraglutide 3.0 mg for patients who had undergone RNYGB or AGB. This is surprising given GLP‐1 levels are known to increase post‐surgery in RNYGB patients but not in AGB patients, and as a GLP‐1 analogue, liraglutide 3.0 mg temporarily increase the circulating levels of GLP‐1 in the body. In contrast to previous research we did not observe less weight loss for patients who had had a gastric sleeve. In fact, patients had similar reductions in weight as of 1‐month post‐initiation of liraglutide 3.0 mg and up to 1 year regardless of the type of surgery they had. Discrepancies in our findings may be due to differences in weight management pharmaceuticals or could be due to power issues given the relatively small number of patients who had undergone sleeve gastrectomy and had data on liraglutide 3.0 mg use for 1 year. Nonetheless, this may suggest that liraglutide 3.0 mg is especially beneficial for patients who have undergone gastric sleeve. As we are the first to study differences in weight loss by type of surgery for patients taking liraglutide 3.0 mg, more research is still necessary to substantiate this theory.

Current guidelines for the use of liraglutide 3.0 mg as a weight management pharmaceutical state that patients should discontinue use of the medication if they have not achieved a weight loss of 5% or more after 4 months of use^5^, ^18^ as patients are unlikely to achieve greater weight loss with continued use. Consistent with these findings, approximately 45% of patients in the current study who discontinued taking liraglutide 3.0 mg by 4 months loss less weight than those who persisted taking the medication. In addition, patients discontinued at a similar rate regardless of the type of surgery they had undergone. This further supports the notion that liraglutide 3.0 mg is equally beneficial for weight loss in patients who have undergone any of the three examined bariatric surgery types. In most lifestyle and pharmaceutical weight loss interventions, maximum weight loss is achieved by 6 months.^19^ However, results of the liraglutide 3.0 mg phase III trial observed continued weight loss post 6 months in subjects who had not had bariatric surgery.^6^ Consistent with these results, patients in the current study continued to lose weight up to 1‐year post‐initiation of liraglutide 3.0 mg. This may suggest that post‐bariatric surgery patients can experience sustained weight reduction with the use of this medication.

According to the liraglutide 3.0 mg monograph, gastrointestinal symptoms are prevalent side effects for patients who have not had bariatric surgery.^5^ Consistent with the product monograph,^5^ and research in post‐bariatric surgery patients,^14^ patients in the current study reported side effects that were primarily gastrointestinal in nature, with nausea being the most commonly reported side effect. Overall, the medication appeared to be well tolerated, with only three patients reporting discontinuing due to adverse events (ie, two patients due to an allergic reaction, and one patient due to pancreatitis). Pancreatitis is a known potential side effect of liraglutide 3.0 mg and may occur in 0.1% to 0.3% of patients.^5^ In the current study, the proportion was slightly higher but this could be attributed to our smaller sample size. Nonetheless, these results suggest that the side effects experienced by patients with bariatric surgery are similar to those reported in non‐bariatric surgery patients taking liraglutide 3.0 mg and supports the notion that this medication may be a safe option to manage excess weight in post‐bariatric surgery patients.

Our study has several strengths and limitations that warrant mentioning. A strength of this study includes being one of the few studies to examine the use of liraglutide 3.0 mg, one of only three available weight loss pharmaceuticals in Canada, in a post‐bariatric surgery population. In addition, this is the first to examine differences in weight loss based on the type of bariatric surgery for patients taking liraglutide 3.0 mg. Patients were also recruited from a publically funded weight management program with a large catchment area increasing the diversity of the sample and the generalization of these results to populations with obesity. Limitations include the retrospective collection of data making us unable to establish a control group. Additionally, we were unable to assess patient medication adherence or dose titration. Currently, 3.0 mg is the only recommended clinical dose of liraglutide for weight management, thus, differences in dose titration or lack of adherence to medication delivery schedule could have resulted in variability in weight loss. Patients date of initiating liraglutide 3.0 mg are based on self‐report at a follow‐up appointment. Current guidelines for the use of liraglutide for weight management recommend discontinuing treatment if the patient is unable to achieve a ≥5% weight loss after 16 weeks of use.^5^, ^18^ As 47% (n = 55) of the patients in this study had taken liraglutide for 4 months or less, our sample may have included patients who are not responsive to liraglutide. Indeed, when patients who have taken liraglutide for 4 months or less are excluded, the proportion of patients who achieve clinically significant weight loss increases from 41.9% to 59.4%. Thus, the inclusion of these patients likely biased our results to the null. Our length of time of follow‐up was variable, we could only verify that 10 of 53 patients had discontinued taking liraglutide 3.0 mg by 4 months.

In summary, in our centre, we found that post‐bariatric surgery patients with insufficient weight loss or excessive weight regain who use liraglutide 3.0 mg were able to achieve a statistically significant weight loss, regardless of the type of bariatric surgery they had undergone. In addition, post‐bariatric surgery patients taking liraglutide 3.0 mg continue to lose weight at 1 year. Further, post‐bariatric surgery patients taking liraglutide 3.0 mg have a similar side effect profile as what is observed in patients without bariatric surgery, with the exception being potentially more severe adverse events reported. These results are promising as current methods for weight management in post‐bariatric surgical patients are limited, and often have greater risks to the patient than the initial procedure itself. More research, using a more rigorous study design, is still necessary to determine the reproducibility of these results and whether certain weight management pharmaceuticals may be more efficacious for post‐bariatric surgery patients than others.

## CONFLICTS OF INTEREST

S.W. is the Medical Director of the Wharton Medical Clinic, and an internal medicine specialist with privileges at Toronto East General Hospital, and Hamilton Health Sciences. S.W. has received payment in the past from Novo Nordisk, Eli Lilly, Janssen, and Astra Zeneca for advisory work. J.L.K. has received funding in the past in the form of research grants from CIHR (#131594). E.K. is currently employed as the Research Coordinator at the Wharton Medical Clinic. R.A.G.C. was previously employed as the Research Coordinator at the Wharton Medical Clinic and is working with Novo Nordisk on research publications. M.L. has no conflicts of interests to report.

## References

[1] Gloy VL , Briel M , Bhatt DL , et al. Bariatric surgery versus non‐surgical treatment for obesity: a systematic review and meta‐analysis of randomised controlled trials. BMJ. 2013;347:f5934. 10.1136/bmj.f5934. PMC3806364 24149519

[2] Buchwald H , Avidor Y , Braunwald E , Jensen MD , Proies W , Fahrbach KSK . Bariatric surgery: a systematic review and meta‐analysis. JAMA. 2004;292(14):1724‐1727. 15479938 10.1001/jama.292.14.1724

[3] Karmali S , Brar B , Shi X , Sharma AM , De Gara C , Birch DW . Weight recidivism post‐bariatric surgery: a systematic review. Obes Surg. 2013;23(11):1922‐1933. 10.1007/s11695-013-1070-4. 23996349

[4] Camacho D , Zundel N. Complications in Bariatric Surgery. https://link.springer.com/content/pdf/10.1007%2F978-3-319-75841-1.pdf. Accessed April 17, 2019.

[5] Novo Nordisk . Saxenda Product Monograph; 2016 http://www.novonordisk.ca/content/dam/Canada/AFFILIATE/www‐novonordisk‐ca/OurProducts/PDF/Saxenda_PM_English.pdf. Accessed March 27, 2017.

[6] Pi‐Sunyer X , Astrup A , Fujioka K , et al. A randomized, controlled trial of 3.0 mg of liraglutide in weight management. N Engl J Med. 2015;373(1):11‐22. 10.1056/NEJMoa1411892. 26132939

[7] Wharton S , VanderLelie S , Sharma AM , Sharma S , Kuk JL . Feasibility of an interdisciplinary program for obesity management in Canada. Can Fam Physician. 2012;58(1):32‐38. PMC3264036 22267637

[8] Wharton S , Liu A , Pakseresht A , et al. Real‐world clinical effectiveness of liraglutide 3.0 mg for weight management in Canada. Value Health. 2018;21:S246 10.1016/j.jval.2018.04.1668. PMC6593982 31062937

[9] Lau DCW , Douketis JD , Morrison KM , Hramiak IM , Sharma AM , Ur E . Canadian clinical practice guidelines on the management and prevention of obesity in adults and children. Can Med Assoc J. 2007;176(8 suppl):s1‐s13. 10.1503/cmaj.061409. PMC1839777 17420481

[10] National Institutes of Health . The Practical Guide: Identification, Evaluation, and Treatment of Overweight and Obesity in Adults. NIH Publication No. 984083; 2000 http://www.nhlbi.nih.gov/guidelines/obesity/ob_gdlns.htm

[11] Brolin RE , Cody RP . Weight loss outcome of revisional bariatric operations varies according to the primary procedure. Ann Surg. 2008;248(2):227‐232. 10.1097/SLA.0b013e3181820cdf. 18650632

[12] Schwartz J , Suzo A , Wehr AM , et al. Pharmacotherapy in conjunction with a diet and exercise program for the treatment of weight recidivism or weight loss plateau post‐bariatric surgery: a retrospective review. Obes Surg. 2016;26(2):452‐458. 10.1007/s11695-015-1979-x. 26615406

[13] Nor Hanipah Z , Nasr EC , Bucak E , et al. Efficacy of adjuvant weight loss medication after bariatric surgery. Surg Obes Relat Dis. 2018;14(1):93‐98. 10.1016/j.soard.2017.10.002. 29287757

[14] Rye P , Modi R , Cawsey S , Sharma AM . Efficacy of high‐dose liraglutide as an adjunct for weight loss in patients with prior bariatric surgery. Obes Surg. 2018;28(11):3553‐3558. 10.1007/s11695-018-3393-7. 30022424

[15] le Roux CW , Welbourn R , Werling M , et al. Gut hormones as mediators of appetite and weight loss after Roux‐en‐Y gastric bypass. Ann Surg. 2007;246(5):780‐785. 10.1097/SLA.0b013e3180caa3e3. 17968169

[16] Dirksen C , Jørgensen NB , Bojsen‐Møller KN , et al. Gut hormones, early dumping and resting energy expenditure in patients with good and poor weight loss response after Roux‐en‐Y gastric bypass. Int J Obes (Lond). 2013;37(11):1452‐1459. 10.1038/ijo.2013.15. 23419600

[17] Stanford FC , Alfaris N , Gomez G , et al. The utility of weight loss medications after bariatric surgery for weight regain or inadequate weight loss: a multi‐center study. Surg Obes Relat Dis. 2017;13(3):491‐500. 10.1016/j.soard.2016.10.018. 27986587 PMC6114136

[18] Novo Nordisk . Liraglutide 3.0 mg for Weight Management NDA 206‐321 Briefing Document; 2014 https://www.fda.gov/downloads/advisorycommittees/committeesmeetingmaterials/drugs/endocrinologicandmetabolicdrugsadvisorycommittee/ucm413318.pdf. Accessed May 15, 2017.

[19] Franz MJ , VanWormer JJ , Crain AL , et al. Weight‐loss outcomes: a systematic review and meta‐analysis of weight‐loss clinical trials with a minimum 1‐year follow‐up. J Am Diet Assoc. 2007;107(10):1755‐1767. 10.1016/j.jada.2007.07.017. 17904936

